# Persistent popliteal lymphatic muscle cell coverage defects despite amelioration of arthritis and recovery of popliteal lymphatic vessel function in TNF-Tg mice following anti-TNF therapy

**DOI:** 10.1038/s41598-022-16884-y

**Published:** 2022-07-26

**Authors:** H. Mark Kenney, Yue Peng, Richard D. Bell, Ronald W. Wood, Lianping Xing, Christopher T. Ritchlin, Edward M. Schwarz

**Affiliations:** 1 Center for Musculoskeletal Research, University of Rochester Medical Center, 601 Elmwood Ave, Box 665, Rochester, NY 14642 USA; 2 Department of Pathology & Laboratory Medicine, University of Rochester Medical Center, Rochester, NY USA; 3 Department of Research, Hospital for Special Surgery, New York, NY USA; 4 Department of Obstetrics and Gynecology, University of Rochester Medical Center, Rochester, NY USA; 5 Department of Neuroscience, University of Rochester Medical Center, Rochester, NY USA; 6 Department of Urology, University of Rochester Medical Center, Rochester, NY USA; 7 Department of Medicine, Division of Allergy, Immunology, Rheumatology, University of Rochester Medical Center, Rochester, NY USA; 8 Department of Orthopaedics, University of Rochester Medical Center, Rochester, NY USA

**Keywords:** Rheumatoid arthritis, Lymphatic vessels

## Abstract

While rheumatoid arthritis patients and tumor necrosis factor transgenic (TNF-Tg) mice with inflammatory-erosive arthritis display lymphatic drainage deficits, the mechanisms responsible remain unknown. As ultrastructural studies of joint-draining popliteal lymphatic vessels (PLVs) in TNF-Tg mice revealed evidence of lymphatic muscle cell (LMC) damage, we aimed to evaluate PLV-LMC coverage in TNF-Tg mice. We tested the hypothesis that alpha smooth muscle actin (αSMA)^+^ PLV-LMC coverage decreases with severe inflammatory-erosive arthritis, and is recovered by anti-TNF therapy facilitated by increased PLV-LMC turnover during amelioration of joint disease. TNF-Tg mice with established disease received anti-TNF monoclonal antibody (mAb) or placebo IgG isotype control mAb therapy (n = 5) for 6-weeks, while wild-type (WT) littermates (n = 8) received vehicle (PBS). Bromodeoxyuridine (BrdU) was also administered daily during the treatment period to monitor PLV-LMC turnover. Effective anti-TNF therapy was confirmed by longitudinal assessment of popliteal lymph node (PLN) volume via ultrasound, PLV contraction frequency via near-infrared imaging of indocyanine green, and ankle bone volumes via micro-computed tomography (micro-CT). Terminal knee micro-CT, and ankle and knee histology were also performed. PLVs were immunostained for αSMA and BrdU to evaluate PLV-LMC coverage and turnover, respectively, via whole-mount fluorescent microscopy. Anti-TNF therapy reduced PLN volume, increased talus and patella bone volumes, and reduced tarsal and knee synovial areas compared to placebo treated TNF-Tg mice (*p* < *0.05*), as expected. Anti-TNF therapy also increased PLV contraction frequency at 3-weeks (from 0.81 ± 1.0 to 3.2 ± 2.0 contractions per minute, *p* < *0.05*). However, both anti-TNF and placebo treated TNF-Tg mice exhibited significantly reduced αSMA^+^ PLV-LMC coverage compared to WT (*p* < *0.05*). There was no correlation of αSMA^+^ PLV-LMC coverage restoration with amelioration of inflammatory-erosive arthritis. Similarly, there was no difference in PLV-LMC turnover measured by BrdU labeling between WT, TNF-Tg placebo, and TNF-Tg anti-TNF groups with an average of < 1% BrdU^+^ PLV-LMCs incorporated per week. Taken together these results demonstrate that PLV-LMC turnover in adult mice is limited, and that recovery of PLV function during amelioration of inflammatory-erosive arthritis occurs without restoration of αSMA^+^ LMC coverage. Future studies are warranted to investigate the direct and indirect effects of chronic TNF exposure, and the role of proximal inflammatory cells on PLV contractility.

## Introduction

Proper lymphatic function and synovial drainage are essential for joint homeostasis. Lymphatic dysfunction with reduced clearance and lymphatic vessel contractility is a well-described contributor to the progression of inflammatory-erosive arthritis in the tumor necrosis factor transgenic (TNF-Tg) mouse model^1,2^. Similarly, rheumatoid arthritis (RA) patients with active disease exhibit reduced lymphatic function compared to healthy controls^3^. The lymphatic system is organized as a lymphatic endothelial cell (LEC) monolayer embedded within the tissues that progressively increases in lymphatic muscle cell (LMC) coverage from pre-collecting to collecting lymphatic vessels that drain into efferent lymph nodes^4^. The collecting lymphatic vessels exhibit forceful spontaneous contractions to propel fluid through the vessel, and are essential for lymphatic homeostasis in the absence of passive fluid flow provided by mechanical forces from adjacent tissue^5,6^. These contractile events also rely on the balance between luminal pressure and vessel tone, mediated in part by LMC coverage, in which vessel tone resists luminal pressure until the resultant axial stress promotes a contraction^7^.

The collecting popliteal lymphatic vessels (PLVs) of TNF-Tg mice demonstrate a progressive decrease in lymphatic contractility and intra-luminal fluid velocity^8–11^. During early stages of inflammatory-erosive arthritis, lymphatic flow is maintained^10^, while the efferent popliteal lymph nodes (PLNs) exhibit a dramatic and prolonged “Expansion” phase. This PLN expansion is associated with intra-nodal lymphangiogenesis, volume expansion, and increased blood flow, noted by both contrast enhanced MRI^12,13^ and power doppler ultrasound^14,15^. Eventually, the PLVs fail to contract, which is associated with PLN “Collapse” where there is a dramatic and sudden reduction in volume and blood flow that occurs concomitant with the onset of severe synovitis and bone erosions in adjacent joints^9,13,15^. These dynamic lymph node changes are associated histopathologically with translocation of CD23^+^/CD21^hi^ B-cells (termed B-cells in inflamed nodes (Bin cells)) from the PLN follicles into the sinuses^12,13^. The translocated Bin cells are thought to mechanically inhibit lymphatic flow and exacerbate arthritis as B-cell depletion therapy both reinstates passive lymph drainage and ameliorates joint disease^10^. Interestingly, PLN collapse and the associated knee flare are asymmetric such that the lower limbs of TNF-Tg mice can be studied independently^9,10,12,13,15–17^. These pathologic collapse mechanisms are notably distinct from reductions in lymph node volume associated with resolution of inflammation, where T-cells exhibit anti-lymphangiogenic properties to regress the expanded lymph nodes towards baseline^18,19^. Importantly, similar dynamic lymph node changes suggestive of pathologic collapse have also been shown in clinical correlate studies of patients with rheumatoid arthritis^20,21^ Detailed ex vivo analysis of PLV contractile function in TNF-Tg mice indicated that deficits in PLV contractility precede the pathogenic processes involved in PLN collapse, and thus may be a driving factor in disease pathogenesis and the onset of severe arthritis^8^. Mechanistically, the B-cell translocation and PLN clogging is thought to be related to stagnation of intra-sinus CXCL13^+^ macrophages from loss of lymphatic contractility^2^, and these cellular processes remain an active area of investigation.

Despite the well-described relationship between reduced lymphatic contractility and arthritis progression, the mechanisms associated with the lymphatic dysfunction and joint inflammation remain largely unknown. Several reports indicate that inflammatory factors (i.e., inducible nitric oxide synthase (iNOS)) produced by accumulated peri-lymphatic immune cells may underlie the reduced lymphatic contractility^8,11,22^, while others posit that direct LMC tissue damage is a pivotal event^9,23^. For instance, ultrastructural imaging revealed a notable loss of LMC structure and integrity associated with reduced PLV contraction frequency and lymphatic clearance in vivo by near-infrared imaging of indocyanine green (NIR-ICG)^9^. Despite this early functional decline, 6-weeks of anti-TNF therapy restored PLV contractions^9^, but this restoration was unable to be directly attributed to LMC recovery by regional ultrastructural analysis. Similarly, immunostaining of pre-collecting synovial lymphatics from TNF-Tg mice for alpha smooth muscle actin (αSMA), a biomarker of LMC coverage, demonstrated a progressive and accelerated reduction in LMC investiture with age^23,24^ relative to typical aging processes^25^. While unique PLV-LMC progenitors have recently been described during postnatal growth and development^26^, it remains unknown whether these precursors are capable of turnover and functional recovery following injury^27^. Herein, we describe our examination of PLV-LMC coverage in TNF-Tg mice with severe inflammatory-erosive arthritis, and evaluation of anti-TNF therapy on PLV-LMC investiture and turnover.

## Results

### Confirmation of effective anti-TNF therapy with amelioration of inflammatory-erosive arthritis in TNF-Tg mice

**Figure 1:**
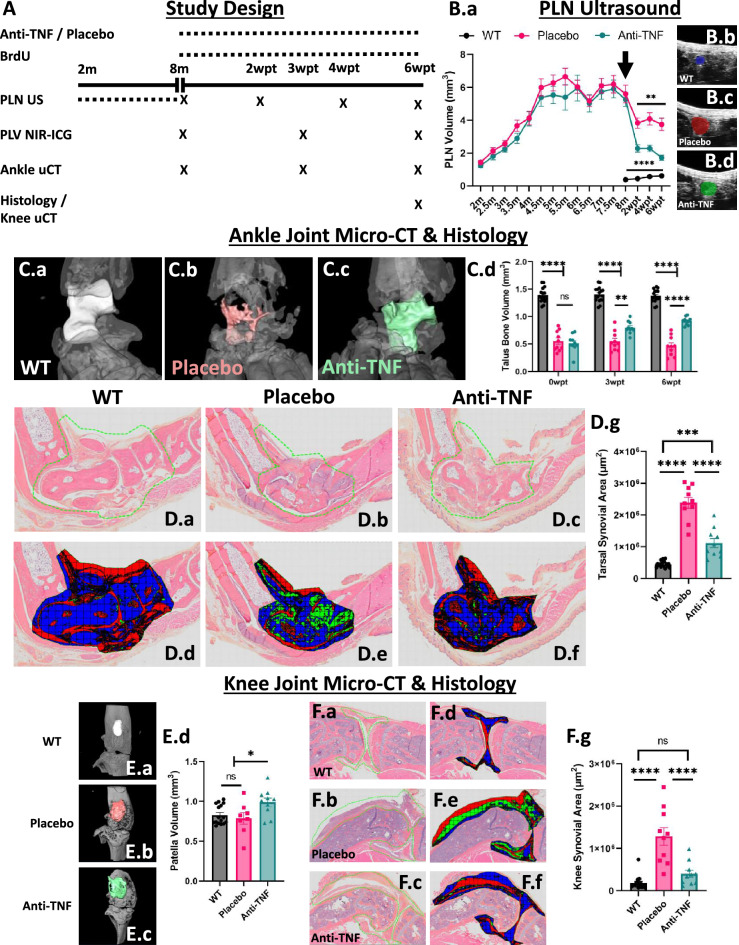
Confirmation of effective anti-TNF therapy with amelioration of inflammatory-erosive arthritis in TNF-Tg mice. The study design is provided to describe the treatment period along with PLN, PLV, and joint measurements to evaluate anti-TNF therapy efficacy **(A)**. PLN volumes quantified by ultrasound were tracked biweekly starting at 2-months of age, and mice were stratified into anti-TNF monoclonal antibody (mAb) or placebo mAb isotype control (n = 5 mice / group) treatment groups based on PLN volumes at 8-months of age. WT littermates (n = 8 mice) received volume-matched vehicle (PBS). Longitudinal measures of PLN volumes showed that WT PLNs had significantly reduced volumes compared to both TNF-Tg groups at the initiation of therapy (**B.a**, arrow), while anti-TNF therapy promoted a greater reduction in PLN volume compared to placebo, as highlighted by the representative PLN cross-sectional B-mode images **(B.b–B.d)**. Similarly, representative images of the talus in the ankle (colored bones) at 6wpt **(C.a–C.c)** demonstrated the significant increase in bone volumes with anti-TNF therapy throughout the treatment period **(C.d)**. Ankle joint recovery was validated by H&E-OG stained sections evaluated for synovial area within the tarsal bone region **(D.a–D.c)** with semi-automated segmentation of synovium (green), adipose tissue / white space (red), and other bone / soft tissue (blue) **(D.d–D.f)**. Quantification showed significantly reduced synovial area in anti-TNF vs placebo treated TNF-Tg mice **(D.g)**. In the knee, representative images of patella bones **(E.a–E.c)** showed significantly increased bone volumes in anti-TNF vs placebo TNF-Tg joints, suggesting increased bone growth **(E.d)**. Resolution of inflammation was confirmed by H&E-OG stained histology **(F.a–F.c**) with segmentation **(F.d–F.f)** demonstrating a significant reduction in synovial area (green) for anti-TNF treated TNF-Tg mice **(F.g)**. Statistics: Two-way ANOVA **(B.a, C.d)** or one-way ANOVA **(D.g, E.d, F.g)** with Tukey’s multiple comparisons; * p < *0.05*, ** p < *0.01*, *** p < *0.001*, **** p < *0.0001*. Sample size: Each datapoint represents an individual limb from WT (n = 8, n = 16 limbs), TNF-Tg placebo (n = 5 mice, n = 10 limbs), and TNF-Tg anti-TNF (n = 5 mice, n = 10 limbs) cohorts. All data is presented as mean ± SEM.

To confidently investigate the αSMA^+^ PLV-LMC coverage in TNF-Tg mice, and the potential for changes in LMC turnover rate with anti-TNF therapy, we first had to confirm that the anti-TNF therapy ameliorated inflammatory-erosive arthritis, as previously described^9,28^. During the 6-week anti-TNF or placebo treatment period, mice were evaluated by PLN ultrasound (biweekly), PLV NIR-ICG imaging (triweekly), and ankle micro-computed tomography (micro-CT, triweekly). Joint histology and knee micro-CT were collected terminally following the 6-week treatment period (Fig. 1A). To match the TNF-Tg mice for assignment to placebo or anti-TNF treatment groups, biweekly longitudinal PLN volume measurements by ultrasound were initiated at 2-months of age to evaluate the transition from PLN expansion (increased and sustained volume) to PLN collapse (reduced volume) as a biomarker for the onset of severe synovitis and bone erosions at ~ 8-months of age in TNF-Tg male mice^13^. Following the initiation of treatment at 8-months of age, the PLN volumes decreased in placebo-treated mice, indicating onset of PLN collapse, while the PLN volumes for anti-TNF treated mice were significantly reduced versus placebo, consistent with effective therapy^28^. The differences in reduced PLN volume between pathologic collapse (i.e., B-cell sinus clogging^2^) and resolution of inflammation (i.e., immune cell mediated sinus regression^18^) may be explained by the different mechanisms of action. The WT PLNs exhibited significantly lower PLN volumes compared to both TNF-Tg treatment groups, as expected^29^ (6-weeks post-treatment (6wpt): WT 0.62 ± 0.22, Placebo 3.8 ± 1.2, and anti-TNF 1.7 ± 0.50 mm^3^; mean ± SD) (Figs. 1B.a–B.d).

Longitudinal in vivo measures of talus bone volumes, a biomarker of arthritis-associated bone erosion in the ankle joints of TNF-Tg mice^17^, showed increased bone volumes by 3wpt in anti-TNF compared to placebo treated TNF-Tg mice. At 6wpt, anti-TNF treated talus bone volumes remained significantly elevated versus placebo showing effective therapy, but did not recover back to WT levels (6wpt: WT 1.4 ± 0.12, Placebo 0.46 ± 0.18, and anti-TNF 0.92 ± 0.071 mm^3^) (Figs. 1C.a–C.d). Similarly, hematoxylin and eosin with orange G (H&E-OG)-stained histology sections of the ankle with semi-automated segmentation of the synovium within the tarsal region showed reduced synovial area for anti-TNF versus placebo treated TNF-Tg mice, although synovial areas remained elevated relative to WT (WT 4.5 × 10^5^ ± 9.9 × 10^4^, Placebo 2.4 × 10^6^ ± 5.4 × 10^5^, and anti-TNF 1.1 × 10^6^ ± 4.5 × 10^5^ μm^2^) (Figs. 1D.a–D.g). Patella bone volumes in the knee by micro-CT were also increased for anti-TNF compared to placebo treated TNF-Tg mice. However, placebo treated patella bone volumes were similar to WT, suggesting that anti-TNF therapy increases bone growth in TNF-Tg mice (WT 0.83 ± 0.12, Placebo 0.79 ± 0.20, and anti-TNF 0.99 ± 0.17 mm^3^) (Figs. 1E.a–E.d). The knee synovial area returned to WT levels in anti-TNF versus placebo treated TNF-Tg mice (WT 1.9 × 10^5^ ± 1.6 × 10^5^, Placebo 1.3 × 10^6^ ± 6.6 × 10^5^, and anti-TNF 4.0 × 10^5^ ± 2.8 × 10^5^ μm^2^) (Figs. 1F.a–F.g). Taken together, measures of PLN volume, joint micro-CT, and joint histology serve as a combinatorial confirmation of effective anti-TNF therapy in this study.

### TNF-Tg PLVs exhibit reduced αSMA^+ ^LMC coverage that persists following anti-TNF therapy

**Figure 2:**
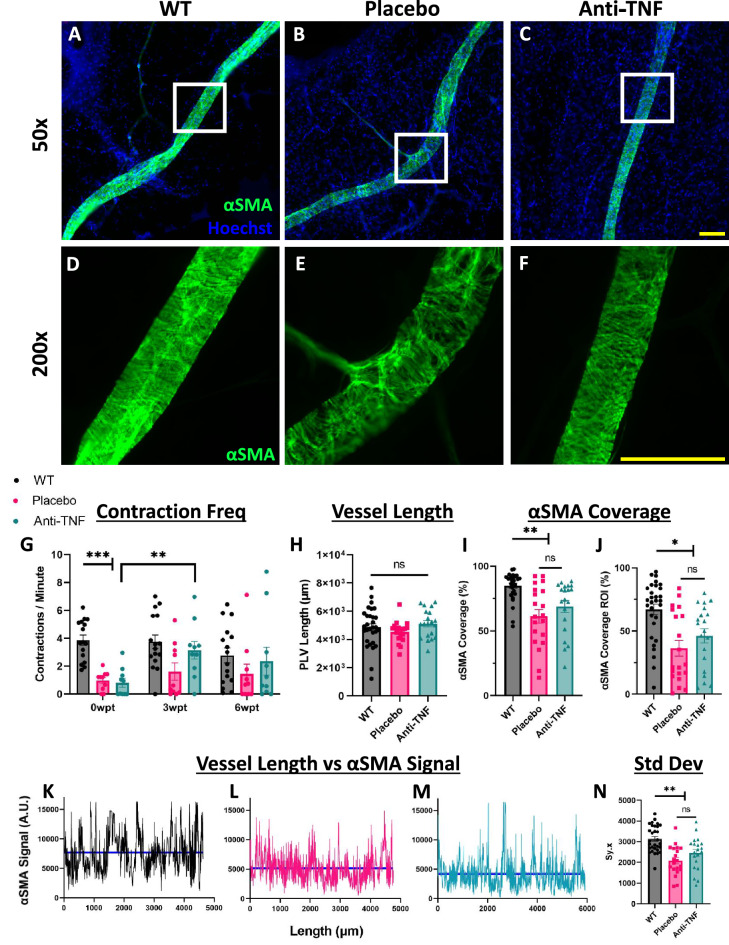
TNF-Tg PLVs exhibit reduced αSMA^+^ LMC coverage that persists following anti-TNF therapy. After 6-weeks of anti-TNF therapy, PLVs were harvested and subjected to whole-mount immunostaining for αSMA (green) with a Hoechst nuclear dye **(A-C)**. High-magnification of whole-mount images (white boxes) illustrate the αSMA^+^ LMC coverage of PLVs from WT **(D)** and TNF-Tg mice treated with placebo **(E)** or anti-TNF **(F)** monoclonal antibodies. To evaluate associated in vivo PLV functional outcomes with the ex vivo histologic measures, contraction frequency was assessed by NIR-ICG imaging and the data for each lower limb is presented **(G)**. PLV length for each lower limb is shown, and the differences between groups were not significant (ns) **(H)**. The % αSMA^+^ LMC coverage was quantified from the whole mount immunofluorescent microscopy images **(I)**. Similar results were obtained via a minimum αSMA region of interest analysis **(J)**, along with assessment of αSMA^+^ LMC signal spatial patterning across the vessel length, as illustrated by traces from representative PLVs (**K–M)** and quantitative analysis of signal variance per length **(N)**. Note the reduction in the mean intensity line (blue horizontal) for both TNF-Tg treatment cohorts vs WT with significantly increased variance around the mean signal line for WT vs TNF-Tg PLVs regardless of therapy. Statistics: Two-way **(G)** and one-way ANOVA **(H, J, N)** with Tukey’s multiple comparisons or Kruskal–Wallis with Dunn’s multiple comparisons **(I)**; * p < *0.05*, ** p < *0.01*, *** p < *0.001*. Sample size: Each datapoint represents average PLV contraction frequency per limb or individual αSMA^+^ PLV-LMC intensity measures from WT (n = 8 mice, n = 16 limbs, n = 30 PLVs), TNF-Tg placebo (n = 5 mice, n = 10 limbs, n = 20 PLVs), and TNF-Tg anti-TNF (n = 5 mice, n = 10 limbs, n = 20 PLVs) cohorts. All data is presented as mean ± SEM. Yellow scale bar = 100 μm **(A–F)**.

Based on the observation that PLV contraction frequency is restored following 6-weeks of anti-TNF therapy in TNF-Tg mice^9^, we hypothesized that LMC coverage is recovered following TNF blockade as a mechanism to explain improved PLV function. We selected αSMA as a biomarker for LMC coverage, as previously described^23,25,27,30^. PLVs were harvested and subjected to whole-mount immunostaining for αSMA (green) following the 6-weeks of effective anti-TNF therapy, as shown in Fig. 1. Representative images of a WT, TNF-Tg Placebo, and TNF-Tg anti-TNF PLV are shown (Figs. 2A–C) with high-magnification images provided to demonstrate the expected αSMA^+^ PLV-LMC coverage in WT compared to the notably reduced signal intensity for both treatment cohorts of TNF-Tg mice (Figs. 2D–F). Interestingly, in vivo functional assays measuring contraction frequency of these PLVs by NIR-ICG imaging showed recovery at 3wpt in anti-TNF treated TNF-Tg mice (0wpt: 0.81 ± 1.0 vs 3wpt: 3.2 ± 2.0 contractions per minute), similar to previous studies^9^, but the recovery was limited and variable across the treatment period (Fig. 2G). Despite these findings, consistent dissections of PLVs between groups (Fig. 2H, ~ 5 mm PLV length) showed significantly reduced αSMA^+^ LMC coverage across the entire PLV length in TNF-Tg mice, which was not affected by anti-TNF therapy (WT 85.0 ± 10.8, Placebo 61.3 ± 23.5, and anti-TNF 68.9 ± 20.4% αSMA^+^ PLV-LMC coverage) (Fig. 2I). Regions of interest with the minimum αSMA signal intensity per PLV were also evaluated for LMC coverage, and a similar reduction in αSMA^+^ LMC investiture for both placebo- and anti-TNF-treated TNF-Tg mice was noted (WT 67.1 ± 23.3, Placebo 36.3 ± 27.8, and anti-TNF 46.2 ± 24.8% αSMA^+^ PLV-LMC coverage) (Figs. 2J & Supplementary Fig. 1). Additional measures of αSMA^+^ LMCs, such as mean and median fluorescent intensity, across the entire PLV and in regions of interest, confirmed the coverage analysis with significantly reduced αSMA signal in both TNF-Tg cohorts vs WT and lack of recovery with anti-TNF therapy (Supplementary Fig. 1).

Apart from global or regional αSMA^+^ PLV-LMC coverage, we also investigated spatial patterning of αSMA signal across the PLV length as adjacent LMCs propagate contractile events across the lymphangion for coordinated contractions^31,32^. Signal patterning was assessed by measuring the standard deviation of signal intensity around the mean as a function of vessel length (Sy.x, where y = signal intensity, x = length) based on well-described regional LMC coverage differences of lymphatic vessels^25^. While WT αSMA^+^ PLV-LMC coverage demonstrated expected high signal variance, both placebo and anti-TNF treated TNF-Tg mice exhibited consistently reduced αSMA signal across the PLV with limited variance around the mean (WT 3136 ± 640, Placebo 2082 ± 702, and anti-TNF 2447 ± 802 Sy.x of artificial units (A.U.) for αSMA signal intensity) (Figs. 2K–N). Overall, these findings indicate that TNF-Tg PLVs exhibit reduced αSMA^+^ LMC coverage, which cannot be restored by 6-weeks of anti-TNF therapy. Moreover, and unexpectedly, αSMA^+^ PLV-LMC coverage is not associated with lymphatic contraction frequency.

### Joint erosions and synovitis are unrelated to αSMA^+^ LMC coverage of PLVs

**Figure 3:**
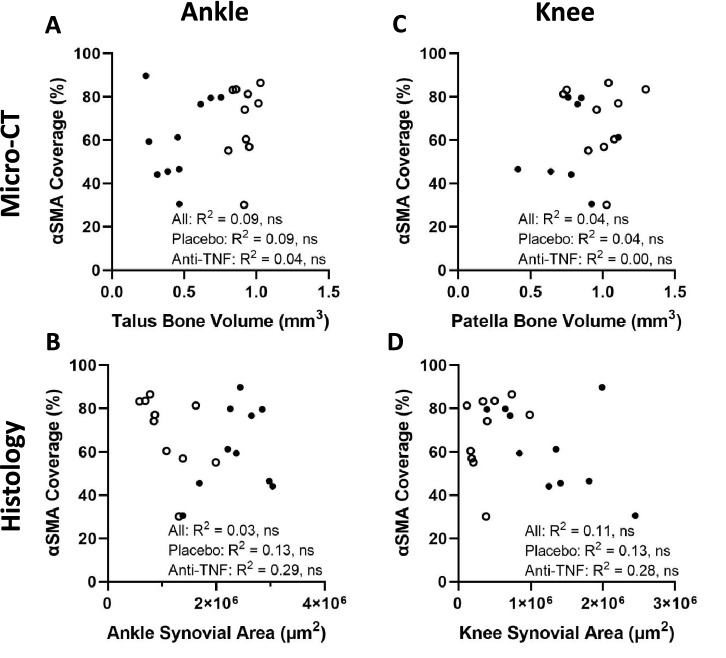
Joint erosions and synovitis are unrelated to αSMA+ LMC coverage of PLVs. To investigate the relationship between joint disease and αSMA^+^ PLV-LMC coverage, we performed linear regression analysis with bone volumes by micro-CT or synovial areas by histology for placebo (closed circles) and anti-TNF (open circles) treated TNF-Tg mice. We found no association between talus bone volumes **(A)** or synovial area in the tarsal region **(B)** of the ankles with the αSMA^+^ PLV-LMC coverage regardless of treatment. Similarly, there was no relationship between patella bone volumes **(C)** or synovial area **(D)** of the knee with αSMA^+^ PLV-LMC coverage for both TNF-Tg treatment cohorts. Statistics: Linear regression analysis with R^2^ and *p*-value provided for placebo and anti-TNF combined (All) or the treatment groups separately; no significance (ns), *p* > *0.05*. Sample size: Each datapoint represents an individual limb with the average αSMA^+^ PLV-LMC coverage of the two PLVs per limb from TNF-Tg placebo and TNF-Tg anti-TNF cohorts (n = 5 mice, n = 10 limbs per group).

To assess the relationship between outcome measures of inflammatory-erosive arthritis and αSMA^+^ PLV-LMC coverage, we performed linear regression analysis for both TNF-Tg placebo and anti-TNF treatment groups together and separately (Fig. 3). First, the bone volumes and synovial areas for each joint were analyzed to evaluate the relationship of these outcome measures as biomarkers of arthritic severity. Talus bone volumes and synovial area in the tarsal region of the ankle were correlated in the combined groups (All, R^2^ = 0.50, *p* < *0.001*). However, patella bone volumes and synovial areas at the knee were unrelated between treatment groups (All, R^2^ = 0.09), suggesting independent pathologic processes at the knee joint (Supplementary Fig. 2). Comparison of arthritis with PLV-LMCs showed no relationship between severity (Placebo, R^2^ = 0.09/R^2^ = 0.13), recovery (anti-TNF, R^2^ = 0.04/R^2^ = 0.29), or combined treatment effects (All, R^2^ = 0.09/R^2^ = 0.03) of talus bone erosions/tarsal synovitis and αSMA^+^ PLV-LMC coverage (Figs. 3A,B). Similarly, αSMA^+^ PLV-LMC coverage showed no relationship with the severity (Placebo, R^2^ = 0.04/R^2^ = 0.00), recovery (anti-TNF, R^2^ = 0.13/R^2^ = 0.28), or combined treatment effects (All, R^2^ = 0.04/R^2^ = 0.11) of patellar erosions/knee synovitis (Figs. 3C,D). These results indicate that anti-TNF therapy ameliorates inflammatory-erosive arthritis via mechanisms independent of αSMA^+^ PLV-LMC coverage.

### WT and TNF-Tg PLV-LMCs exhibit limited and unchanged turnover by 8-months of age

**Figure 4:**
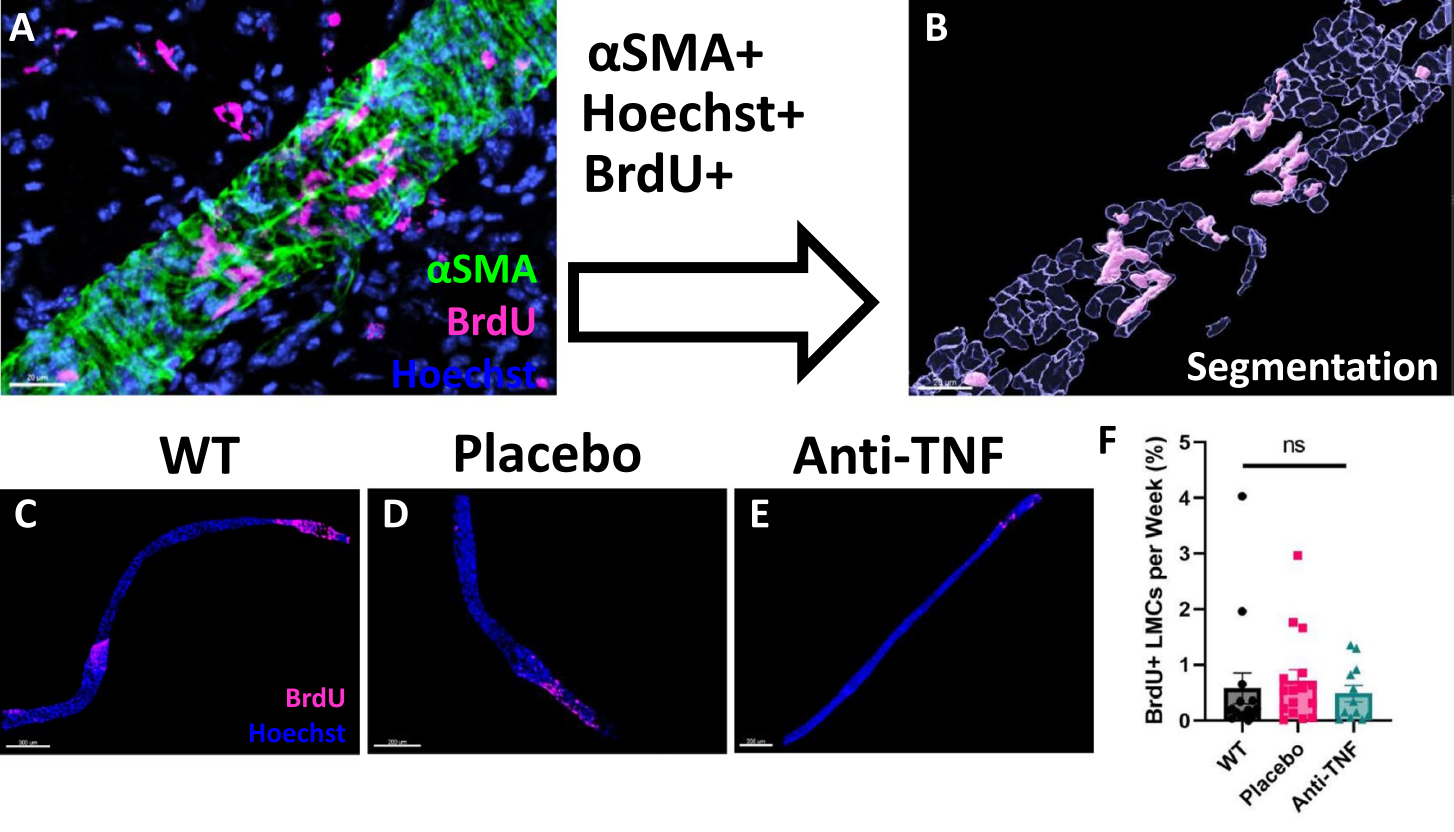
WT and TNF-Tg PLV-LMCs exhibit limited and unchanged turnover by 8-months of age. Along with the placebo and anti-TNF treatments, BrdU was administered daily for 6-consecutive weeks to evaluate LMC turnover during the treatment period. Following the 6-week period, PLVs from WT and TNF-Tg mice were harvested and immunostained for αSMA (green) and BrdU (pink) with a Hoechst nuclear dye (blue). For cellular colocalization analysis, confocal stacks across the entire length and depth of the whole-mounted PLVs were collected. Imaris software was used to visualize total Hoechst^+^ and Hoechst^+^/BrdU^+^ nuclei masked within αSMA^+^ LMCs with a high-magnification image of an anti-TNF treated TNF-Tg PLV provided as a representative image **(A)**. The nuclei were then segmented as individual cells to quantify total Hoechst^+^ LMC nuclei (transparent blue objects) and Hoechst^+^/BrdU^+^ LMC nuclei (solid light pink objects) that contributed to the vessel during the treatment period **(B)**. Representative low-magnification images of WT **(C)** and TNF-Tg PLVs treated with placebo **(D)** or anti-TNF **(E)** therapy are shown with no significant difference in the proportion of αSMA^+^/BrdU^+^ LMCs per week **(F)**. Statistics: One-way ANOVA with Tukey’s multiple comparisons; no significance (ns), *p* > *0.05*. Sample size: Each datapoint represents an individual PLV from WT (n = 4 mice, n = 15 PLVs), TNF-Tg placebo (n = 4 mice, n = 16 PLVs), and TNF-Tg anti-TNF (n = 3 mice, n = 12 PLVs) cohorts. All data is presented as mean ± SEM.

Based on our findings that PLV-LMCs derive from unique progenitor origins compared to adjacent venous vascular smooth muscle cells during postnatal development^26^, we next evaluated PLV-LMC turnover in WT versus TNF-Tg mice, along with the effects of anti-TNF therapy, by bromodeoxyuridine (BrdU) labeling. Confocal image stacks across the entire length and depth of the whole-mounted PLVs were visualized using Imaris software, and all LMC-associated Hoechst^+^ (blue) and BrdU^+^ (pink) nuclei within αSMA^+^ cells (green) were analyzed (Fig. 4A). The αSMA^+^ PLV-LMC-associated Hoechst^+^ and BrdU^+^ nuclei were quantified as a turnover rate of BrdU^+^/Hoechst^+^ colocalization relative to total Hoechst^+^ nuclei (Fig. 4B). Representative images of PLVs from WT (Fig. 4C) and TNF-Tg mice treated with placebo (Fig. 4D) or anti-TNF (Fig. 4E) therapy are provided, and quantification of LMC turnover rate demonstrated no change between groups with an average of < 1% BrdU^+^ PLV-LMCs per week of treatment (Fig. 4F). Also note the regional pattern of BrdU^+^ LMC incorporation (late growth/maintenance), relative to the significantly increased rate and widespread pattern of LMC contribution to the PLVs in neonatal WT mice, shown in Supplementary Fig. 3 (early growth/development) (Adult WT 0.58 ± 1.1, Placebo 0.71 ± 0.79, and anti-TNF 0.48 ± 0.50 versus neonatal WT 21.4 ± 15.0 BrdU^+^ LMCs per week, *p* < *0.0001* for neonatal vs. all adult cohorts). Isotype control staining was also used to validate the BrdU labeling on the experimental vessels (n = 2 PLVs, 1 WT, 1 anti-TNF), where approximately 0.17 ± 0.02% BrdU^+^ LMCs per week represent non-specific staining. To confirm effective in vivo BrdU labeling in the treatment groups, associated PLN histology sections were immunostained for BrdU, which confirmed BrdU incorporation compared to the isotype-stained control of the adjacent section (Supplementary Fig. 3). These findings demonstrate that the LMC incorporation rate is low in both WT and TNF-Tg adult mice, and thus the reduction of αSMA^+^ PLV-LMC coverage in TNF-Tg cohorts is independent of changes in LMC turnover.

## Discussion

In this work, we found that direct LMC damage may not be responsible for the reduced lymphatic function and onset of severe inflammatory-erosive arthritis in TNF-Tg mice. Alternative mechanisms, such as chemical inhibition of lymphatic contractility through the accumulation of peri-lymphatic inflammatory immune cells^22^ or interactions with adjacent LECs^11^, ought to be investigated in future studies. Of particular relevance, NOS inhibition is effective in recovering lymphatic contractility in some studies^8,11,33^,while recent investigation of global genetic iNOS ablation demonstrated limited effects on lymphatic function and arthritis progression in TNF-Tg mice^29^. The LEC is also an essential contributor to collecting lymphatic vessel contractile mechanisms^5^, and can directly regulate LMC function through endothelial NOS (eNOS) and iNOS production^11^. Along with damaged LMCs identified by ultrastructural imaging, the LECs were also found to be apoptotic in the PLVs of TNF-Tg mice with severe arthritis^9^. These findings suggest that the cellular contribution to lymphatic function is a complex and dynamic phenomenon where multiple cell types (i.e., LMCs, LECs, macrophages, mast cells^34,35^, etc.) may be involved, and future investigation into the interactions of these peri-lymphatic cells during inflammation and therapy is warranted.

While our study provides novel findings that αSMA^+^ PLV-LMC coverage is reduced in TNF-Tg mice with severe arthritis, and that this reduced coverage persists without turnover or recovery following effective anti-TNF therapy, our study has notable limitations. A primary limitation is the reliance on αSMA as a biomarker of PLV-LMC coverage. While αSMA has been utilized as a biomarker of LMC coverage that associates with lymphatic function in previous studies^23,25,27^, other proteins (i.e. myosin heavy chain 11 (Myh11))^36^ or transcriptional changes (i.e. *Zbtb16*)^22^ may have recovered during the treatment period associated with amelioration of arthritis. In addition, based on our unsuccessful prospero homeobox 1 (Prox1) staining (as previously performed^26^) and lack of *bona fide* valve associated markers (i.e., integrin-α9^37^), we were limited in our capacity to analyze specific PLV regions (i.e., valve vs non-valve). Despite this limitation, we evaluated the standard deviation of the αSMA fluorescent intensity across the length of the vessel, and determined that the standard deviation decreased in both TNF-Tg groups compared to WT. The decrease in standard deviation suggests a uniform reduction in αSMA signal that is not particular to any region of the PLV. In contrast, if a particular region had been differentially affected (i.e., pre- or post-valve areas) we would have expected an increase in standard deviation of fluorescent intensity across the vessel length. However, a formal evaluation of LMC recovery in the various PLV anatomical regions ought to be considered in future investigation. To evaluate other potential mechanisms of LMC recovery with anti-TNF therapy, or selective recovery of potential location-dependent LMC subpopulations (i.e., valves), future single-cell RNA-sequencing studies of TNF-Tg PLV-LMCs treated with anti-TNF therapy are also warranted.

In addition, since anti-TNF therapy affects both the lymphatic system and the joint simultaneously, the ability to evaluate the direct relationship between lymphatic contractility and arthritis may be a limitation of this study. For future experimentation, strategies to specifically modulate LMC coverage to assess the effect of arthritic progression ought to be considered. As an example, genetic models that inhibit LMC recruitment during development (i.e., *Prox1*^CreER^ x platelet derived growth factor subunit B (*Pdgfb*)^Flox/Flox^ mice^30^) crossed into TNF-Tg mice would allow for a targeted reduction of LMC coverage to determine the effects on arthritis. In our study, the lack of association between the arthritis and the αSMA^+^ PLV-LMCs may be due to differential rates of tissue recovery with TNF inhibition, while these factors could be related at earlier stages of disease. Similarly, the evaluation of TNF-Tg mice with severe arthritis may have reached a ceiling of disease activity where the relationship with αSMA^+^ PLV-LMC coverage was unable to be adequately assessed.

However, our findings also corroborate previous work suggesting LMC damage is irreversible and unable to completely recover following injury. A recent study demonstrated that in skin and soft tissue infections with *Staphylococcus aureus*, that LMC coverage was reduced even out to 260 days post infection, which suggested a chronically sustained LMC depletion. In contrast to our work investigating αSMA levels by immunostaining, the authors utilized an αSMA-DsRed mouse model that measures cells with *Acta2* (αSMA) gene expression by presence of a red fluorescent protein^27^. Along with the sustained reduction in αSMA^+^ PLV-LMC coverage, we further demonstrated that there was no change in PLV-LMC turnover by BrdU labeling in WT and TNF-Tg mice treated with placebo and anti-TNF therapy. Although the PLV-LMC incorporation rate in adult mice was low (< 1% per week), PLV-LMC contribution was present, suggesting that a small proportion of new LMCs continue to differentiate from existing progenitors (assuming LMCs are post-mitotic), similar to neonatal development^26^.

Here we identify continued αSMA^+^/BrdU^+^ LMC incorporation onto PLVs throughout adulthood, which to our knowledge has not been previously reported. The αSMA^+^/BrdU^+^ LMCs notably tended to localize to the terminal ends of the PLVs, suggesting a pattern of growth as opposed to replacement of senescent or otherwise damaged cells. However, the interpretation of the data is limited by the potential for artifactual identification of αSMA^+^/BrdU^+^ LMCs. As we had technical problems with achieving successful Prox1 staining despite previous success^26^, we were not able to negatively exclude possible Prox1^+^/BrdU^+^ LECs from the analysis. In addition, while we masked our analysis within αSMA^+^ stain to exclude peri-lymphatic immune cells outside the PLV, we were unable to definitively exclude immune cells trapped within the lumen of the PLV. To overcome these limitations, we performed confocal microscopy across the entire length and depth of the PLVs with a Z-axis step size of 1.417 μm, while the short-axis of an LMC is > 1.417 μm in length based on previous transmission electron microscopy images^9^. We also performed isotype control staining of experimental vessels of both the PLVs and PLNs from the experimental mice that further confirmed true BrdU labeling. Together, these findings suggest that LMCs continue to incorporate onto growing ends of adult PLVs, while the capacity for replacement of senescent or otherwise damaged cells remains unclear.

An alternative explanation for the lack of LMC recovery following injury that we observed may be a reduction in LMC differentiation and recruitment signals, as opposed to a limitation in the available supply of LMC progenitors. For example, PDGFB^30^ and Reelin / monocyte chemoattractant protein 1 (MCP-1)^38^ signaling from LECs are known to be involved in LMC recruitment to lymphatic vessels during development, and these pathways may not be as robust in adult mice following injury. Even during aging without acute injury, collecting lymphatic vessels exhibit a progressive reduction in LMC coverage^25^, suggesting insufficient LMC incorporation relative to LMC decline. Thus, further investigation into the cellular mechanisms of LMC turnover and/or recruitment in adult and aging collecting lymphatic vessels may reveal therapeutic strategies to promote effective LMC recovery following injury in future studies.

## Methods

### Ethical approval

All animal experiments were approved by the University Committee for Animal Resources at the University of Rochester Medical Center within an AAALAC accredited vivarium. The experiments were performed in accordance with the associated relevant guidelines and regulations for working with live vertebrate animals. Thus, our animal protocols comply with the animal ethical principles under which *Scientific Reports* operates. The animal experiments are also reported in accordance with the Animal Research: Reporting of In Vivo Experiments (ARRIVE) guidelines.

### Mouse models

TNF-Tg mice (3647 line)^39–41^ were initially acquired from Dr. George Kollias and have since been maintained at the University of Rochester. The TNF-Tg mice were bred as heterozygotes, and WT littermates were used as controls (C57BL/6 genetic background). Only male mice were used experimentally due to early female mortality^16^. Sample sizes were determined based on previous studies of anti-TNF treatment in TNF-Tg mice to detect amelioration of arthritis (i.e., synovitis by histology) and lymphatic pathology (i.e., PLV contractions and PLN volume)^9,28^, and each limb was considered an independent datapoint due to the asymmetry of TNF-Tg arthritis, where joints of one limb may experience arthritic flare while the other does not in a single animal^9,10,12,13,15–17^. The asymmetry of the TNF-Tg arthritis was confirmed quantitatively by comparing real limb pairs with randomized limb pairs. The talus bone volume measurements showed that real limb pairs were equivalent to randomly assigning limb pairs, supporting the use of individual limbs as independent variables (Supplementary Fig. 4). A total of 22 mice were used in this study. Two TNF-Tg mice died within 2-weeks of treatment initiation, one placebo and one anti-TNF treated mouse; thus, measures from both of these mice were excluded from the study. For all in vivo longitudinal outcome measures, mice were anesthetized with 1–3% isoflurane. All mice were euthanized with a lethal dose of ketamine/xylazine cocktail (intraperitoneal) followed by cervical dislocation. Genotyping was performed using the following primer sequences:

- TNF-Tg Forward: 5`-TAC-CCC-CTC-CTT-CAG-ACA-CC-3`.
- TNF-Tg Reverse: 5`-GCC-CTT-CAT-AAT-ATC-CCC-CA-3`.

### Treatments

TNF-Tg mice were matched into anti-TNF (CNTO12; Janssen, J&J) or placebo IgG1 isotype control (CNTO151; Janssen, J&J) monoclonal antibody treatment groups based on PLN volume by ultrasound at 8-months-old. Male TNF-Tg mice at 8-months-old approximate the age when PLN collapse occurs and PLN volume serves as a biomarker to estimate the severity of inflammatory-erosive arthritis^9,13–15^. To reduce the potential for confounding, all animals were housed in similar caging conditions, and no animals were housed individually. All animals received their treatments at the same time during the treatment period. TNF-Tg anti-TNF (n = 5 mice) and placebo (n = 5 mice) cohorts were treated for 6-consecutive weeks (i.p., 10 mg/kg/week) as the time-period shown previously to recover lymphatic function^9^. WT littermates (n = 8 mice) were treated with matched volume per weight vehicle PBS. During the 6-week treatment period, all mice were also treated with BrdU (i.p., 0.1 mg/g/day; 5 days on, 2 days off each week), as previously described^26^. To confirm effective BrdU administration and immunostaining, WT neonatal mice (n = 2) were treated with BrdU (i.p., 0.1 mg/g/day) for 1–2 weeks starting at P21 during a phase of rapid animal growth.

### Ultrasound of popliteal lymph nodes

Mice were anesthetized with 1–3% isoflurane and imaged using a Vevo 3100 ultrasound system with the MX700 probe (Fujifilm, Toronto, ON, Canada). Depilatory cream was used to remove the fur on the posterior aspect of both hindlimbs, and mice were placed in the prone position on a temperature-controlled platform set at 37 °C. Hindpaws were secured with adhesive tape to identify the PLNs posterior to the knee, and B-mode images were collected as stacks across the PLN volume. Image stacks (.tif) were then imported into Amira software as luminance image types (v2020.2; ThermoFisher Scientific, FEI, Hillsboro, OR, USA; https://www.thermofisher.com/in/en/home/electron-microscopy/products/software-em-3d-vis/amira-software.html). The lasso tool with auto-trace was used to segment multiple sections of the PLN, which were connected using the interpolate function for volume quantification, as previously described^15,29^. Ultrasound was performed biweekly starting at 2-months-old in the TNF-Tg cohorts, and continued through the 6-week treatment period for all WT and TNF-Tg treatment groups.

### Near-infrared imaging of popliteal lymphatic vessels

NIR imaging was performed using a custom-built system, as previously described^29^.Mice were initially anesthetized with 3% isoflurane, and imaged on 1–2% isoflurane as needed to maintain anesthesia. Careful attention to isoflurane dose was essential for consistent imaging given the known inhibitory effects of isoflurane on lymphatic contraction frequency^42^. For imaging, the mice were placed in the prone position on a temperature-controlled surface set to 37 °C. Depilatory cream was used to remove the fur on the posterior aspect of the hindlimbs. ICG (Millipore Sigma, Cat# 1,340,009) was then administered into the plantar aspect of both hindpaws (intradermal, 10 μL at 0.1 μg/μL), and the hindpaws were secured using adhesive tape. Mechanical pressure was applied to the hindpaw, and the ICG was allowed to absorb into the PLVs for 5 min before imaging. Images were collected for 10 min per limb and image stacks (.tif) were then imported into Fiji (ImageJ, v1.53f51). PLV contractions were manually quantified for the two PLVs in each limb, and contraction frequency was measured as an average of the two PLVs per minute. Analysis was performed blinded with random 4-digit codes assigned to each limb, and identity was revealed to the observer after analysis. Two WT limbs had normal anatomical variants with only one PLV, and for these limbs contraction frequency was a measure of only the single PLV. PLV contraction frequency was measured prior to treatment at 8-months-old then subsequently at 3- and 6-weeks post treatment.

### Joint micro-CT

For ankle micro-CT, mice were anesthetized with 1–3% isoflurane and imaged lying on their left side in a Derlin plastic and clear acrylic tube for 30–45 min. The hindpaws were secured together with adhesive tape and surrounding foam. The knee micro-CT datasets were acquired ex vivo following soft tissue removal and at least 1-day of fixation with 10% neutral buffered formalin (NBF). Datasets were collected using a VivaCT 40 (Scanco Medical, Bassersdorf, Switzerland) with the following imaging parameters: 55 kV, 145 μA, 300 ms integration time, 2048 × 2048 pixels, 1000 projections over 180°, resolution 17.5 μm isotropic voxels. The talus in the ankle and the patella in the knee were segmented in Amira software as biomarkers of arthritis, as previously described^17,43^. Two patellas from the TNF-Tg placebo group were lost during tissue harvest, and thus were not analyzed.

### Joint histology and lymphatic immunostaining

After the 6-weeks of treatment at 9.5-months-old, the mice were euthanized for PLV harvest and immunostaining, as previously described^22,26^. Briefly, mice were administered a lethal dose of ketamine/xylazine cocktail (intraperitoneal), and Evan’s Blue dye (Millipore Sigma Cat# E2129) was injected intradermal into both hindpaws to visualize the PLVs. Depilatory cream was used to remove the fur on the posterior aspect of the hindlimbs, and then the overlying skin was removed. The PLVs were carefully harvested away from or along with the adjacent superficial saphenous vein and placed in PBS. The PLVs were then fixed with 10% NBF for 30 min at room-temperature (RT), washed 3 × 10 min in 1 × TBS (Bio-Rad Cat# 1,706,435)/0.1% Triton X-100 (Millipore Sigma Cat# X100) at RT, and then permeabilized in 1 × TBS / 0.3% Triton X-100 overnight at 4 °C. For the BrdU labeling, PLVs were incubated in 2 M hydrochloric acid (Millipore Sigma Cat# 320,331) for 1 h at RT, 0.1 M Pierce 20X Borate Buffer (ThermoFisher Scientific Cat# 28,341) for 30 min at RT, and washed 3 × 10 min in 1 × TBS / 0.1% Triton X-100. Blocking was performed with 5% normal goat serum (NGS; ThermoFisher Scientific Cat# 50062Z)/1 × TBS/0.3% Triton X-100 for 1 h at RT then the primary antibody was diluted in 5% NGS/1 × TBS / 0.3% Triton X-100 overnight at 4 °C. Both the mouse anti-αSMA (AlexaFluor 488 conjugate; ThermoFisher Scientific Cat# 53-9760-82) and rat anti-BrdU (AlexaFluor 647 conjugate, Abcam Cat# ab220075) antibodies were diluted at 1:100, as previously described^26^. To validate the BrdU labeling, rat IgG2a isotype control antibodies were also applied to a subset of samples (AlexaFluor 647 conjugate, ThermoFisher Scientific Cat# 51-4321-81, 1:100 dilution). As we have previously described^26^, we also applied rabbit anti-Prox1 primary antibody overnight at 4 °C (AngioBio Cat# 11-002P, 1:100 dilution) and goat anti-Rabbit IgG AlexaFluor 555 secondary antibody diluted in 5% NGS / 1 × TBS / 0.1% Triton X-100 for 2 h at RT (ThermoFisher Scientific Cat# A-21428, 1:400 dilution), but achieved non-uniform and insufficient staining for analysis, and thus was omitted. After 3 × 10 min washes in 1 × TBS/0.1% Triton X-100 following antibody incubation, the PLVs were mounted on a microscope slide with one drop of both ProLong Gold Antifade Mountant (ThermoFisher Scientific Cat# P36930) and NucBlue Live ReadyProbes Reagent (Hoechst 33,342 formulation; ThermoFisher Scientific Cat# R37605). The PLVs were then imaged using a VS120 Slide Scanner for αSMA coverage analysis and Nikon A1R HD confocal for BrdU colocalization analysis.

The PLNs were harvested and fixed in 10% NBF for 2 h then processed for paraffin-embedding. Sections at 5 μm were collected, and the day before staining were incubated at 60 °C overnight. The tissue was then dewaxed and rehydrated in graded xylenes / alcohols, and antigen retrieval was performed for 2 h in Coplin jars submerged in simmering water using Citrate-Based Antigen Unmasking Solution (1:100 dilution; Vector Laboratories Cat# H-3300). Once cooled, the PLNs were processed for BrdU immunostaining similar to the PLVs, but with permeabilization using 1 × TBS/0.5% Triton X-100 for 20 min and blocking for 40 min. The PLNs were imaged using a VS120 Slide Scanner.

The ankle and knee joints were harvested and fixed in 10% NBF for 3 days, decalcified in Webb-Jee 14% EDTA solution for 1 week, and then processed for paraffin-embedding. Sections at 5 μm from 3 levels were collected, and stained with H&E-OG. The joint sections were imaged using a VS120 Slide Scanner, and the images (.vsi) were then imported into Visiopharm software (v2021.07; Horsholm, Denmark; https://visiopharm.com). A semi-automated color segmentation protocol was developed in Visiopharm to segment synovial area within a manually-contoured region of interest. The quantification of synovial area for each sample was determined by averaging the values of a section from each of the 3 levels. Ankle synovial area was measured within the joints of the tarsal bones, from the distal tibia to the proximal metatarsals, and tenosynovitis associated with the nearby tendons was excluded.

### Popliteal lymphatic vessel image analysis

Slide scanned images of the PLVs (.vsi) were analyzed in Fiji, and opened with the Olympus Viewer plugin. The FITC channel representing the αSMA stain was manually contoured around the edges of all WT PLVs for each treatment cohort first to measure the median αSMA signal intensity within the vessel boundary. The median value of the median signal intensity from the WT vessels was halved and used as a set threshold to measure αSMA coverage. Each vessel from all treatment groups were then manually contoured with the set threshold applied. Area fraction of αSMA signal above the intensity threshold relative to total vessel area was quantified as αSMA coverage. To identify regions of interest with the lowest αSMA signal intensity, the lookup table was changed to “Fire” and minimum signal intensity was validated through visualization using the Interactive 3D Surface Plot plugin. Once the region with minimum αSMA signal intensity was identified, a constant 500 × 500 μm square was centered on the region, and the portion of the PLV within the square was manually contoured to measure αSMA coverage. Supplementary Fig. 1 provides details on the LMC coverage analysis, and identification of minimum αSMA signal intensity regions across the PLV. Vessel length was quantified using the segmented line tool through the center of the PLV. This segmented line was also used to measure αSMA signal intensity as a function of distance to assess spatial patterning of αSMA across the PLV length. The outcomes of αSMA signal intensity per unit length were imported into GraphPad Prism (v9.3.1; San Diego, CA, USA; https://www.graphpad.com) to measure the standard deviation (Sy.x) of signal intensity (y) as a function of length (x) around the mean signal intensity by non-linear regression analysis.

To evaluate αSMA^+^/Hoechst^+^/BrdU^+^ colocalization, confocal image stacks (.nd2) were collected across the entire length and depth (Z-axis step size of 1.417 μm) for a majority of the PLVs. BrdU labeling was assessed in all PLVs by epifluorescent imaging first, but αSMA^+^/BrdU^+^ LMC localization could not be adequately analyzed without the added Z-dimension of confocal microscopy. A representative subset of the PLVs were imaged with confocal microscopy for this purpose; all PLVs were not evaluated due to technical limitations of imaging time (~ 4 h/PLV) and shared resource availability. The datasets were imported into Imaris software (v9.5.0; Oxford Instruments, Abingdon, United Kingdom; https://imaris.oxinst.com), and analyzed using semi-automated workflows. To accommodate the large file size, many vessels were cropped in half and analyzed separately. First, a surface representing the PLV was generated by manually contouring the outer edge of the vessel (Contour tab: manual, preserve features) and copied across the depth of the PLV image stack. The αSMA channel was then masked within the PLV contour. A surface representing the αSMA^+^ PLV-LMCs was segmented using a set threshold, and then the Hoechst channel was masked within the αSMA^+^ LMCs. The resultant Hoechst^+^ LMC nuclei were segmented as another surface using a set threshold, and split into individual nuclei using a seed point diameter of 3.50 μm. Objects of low quality and/or volume were removed. The BrdU channel was then masked within the Hoechst^+^ nuclei, and segmented as another surface using a set threshold and seed point diameter of 3.50 μm. For quality control, objects with low signal intensity (filtered by median) and/or volume were removed. The total number of BrdU^+^/Hoechst^+^ relative to Hoechst^+^ nuclei within the αSMA mask was used to quantify the LMC turnover rate. One-half of 4 PLVs (1 WT, 2 TNF-Tg placebo, and 1 TNF-Tg anti-TNF) was excluded due to imaging and/or analysis artifacts where background signal generated unclear results.

### Statistics

All statistical analysis, such as two-way or one-way ANOVA with Tukey’s multiple comparisons, non-parametric Kruskal–Wallis with Dunn’s multiple comparisons, Shapiro–Wilk test for normality, and linear or non-linear regressions, was performed in GraphPad Prism. As αSMA coverage across each full vessel was found to have a significantly different distribution from normal by the Shapiro-Wilks test for the WT and anti-TNF groups (** p < *0.01*), this comparison was analyzed using the non-parametric Kruskal–Wallis test (Fig. 2I).

## Supplementary Information

Supplementary Information.

## Data Availability

All data will be made available upon reasonable request. Please contact the corresponding author, EMS, for data availability.
