# Supplementary material for: Persistent popliteal lymphatic muscle cell coverage defects despite amelioration of arthritis and recovery of popliteal lymphatic vessel function in TNF-Tg mice following anti-TNF therapy
Source: Sci Rep. 2022 Jul 26;12:12751. doi: 10.1038/s41598-022-16884-y (PMC9325893; doi:10.1038/s41598-022-16884-y)
Supplement: Supplementary file 1 — Supplementary Information. [file 41598_2022_16884_MOESM1_ESM.docx]

**Supplementary Materials**

**Persistent Popliteal Lymphatic Muscle Cell Coverage Defects Despite Amelioration of Arthritis and Recovery of Popliteal Lymphatic Vessel Function in TNF-Tg Mice Following Anti-TNF Therapy**

H. Mark Kenney^1,2^, Yue Peng^1,2^, Richard D. Bell^3^, Ronald W. Wood^1^, Lianping Xing^1,2^, Christopher T. Ritchlin^1,4^, Edward M. Schwarz^1,2,4,5,*^

^1^Center for Musculoskeletal Research, University of Rochester Medical Center, 601 Elmwood Ave, Box 665, Rochester, NY, 14642, USA.

^2^Department of Pathology & Laboratory Medicine, University of Rochester Medical Center, Rochester, NY, USA

^3^Department of Research, Hospital for Special Surgery, New York, NY, USA

^4^Department of Medicine, Division of Allergy, Immunology, Rheumatology, University of Rochester Medical Center, Rochester, NY, USA

^5^Department of Orthopaedics, University of Rochester Medical Center, Rochester, NY, USA

* Corresponding Author, [Edward_Schwarz@URMC.Rochester.edu](mailto:Edward_Schwarz@URMC.Rochester.edu)


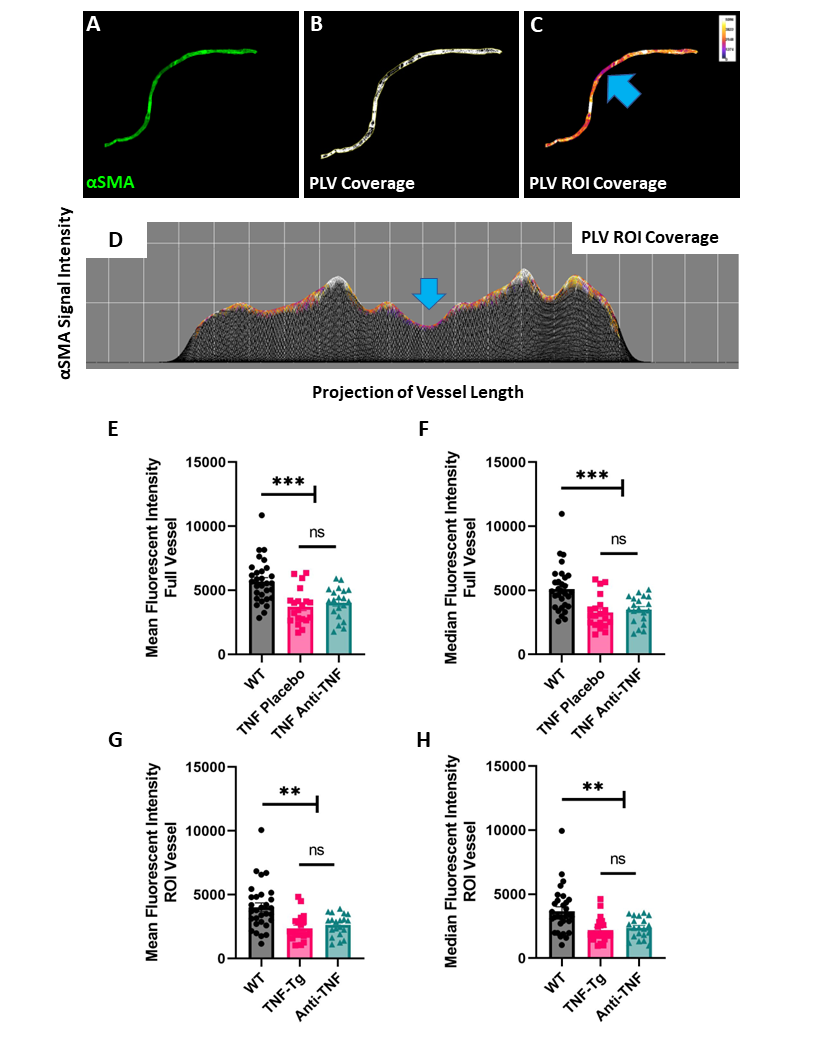
**Supplementary Figure 1. Quantification strategy for global and regional αSMA^+^ PLV-LMC coverage.** The αSMA expression (green) from a representative placebo-treated TNF-Tg PLV is shown **(A)**. A set threshold based on ½ the WT median αSMA signal intensity was used to generate a binary representation of the αSMA expression (white), and LMC coverage was quantified as a percentage of vessel area **(B)**. To evaluate the LMC coverage in a region of interest, we visualized the αSMA expression shown in **A** as a heatmap to localize regions of the PLV images with the lowest αSMA signal intensity **(C)** confirmed by 3D projections of αSMA signal vs vessel length **(D)** (blue arrows). A constant 500 x 500 μm square was centered on the region with the lowest signal intensity, and a set threshold of αSMA coverage per vessel area was similarly quantified as in **B**. Mean and median fluorescent intensity across the length of the vessel and in regions of interest with the lowest signal intensity were compared between the groups to validate the coverage measurements **(E-H)**. Statistics: One-Way ANOVA with Tukey’s Multiple Comparisons; *** p < 0.01, *** p < 0.001* **(E-H)**.


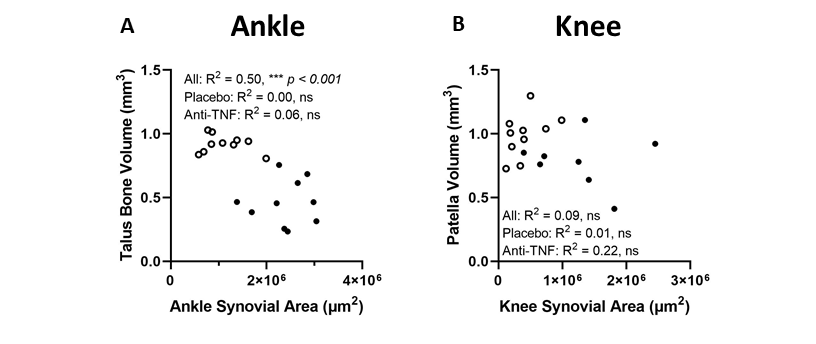


**Supplementary Figure 2. Relationship between synovial areas and bone volumes in arthritic joints from TNF-Tg mice.** To evaluate the dependency of related synovial areas and bone volumes as biomarkers of inflammatory-erosive arthritis, we performed linear regression analysis between the histology and micro-CT outcome measures of placebo (closed circles) or anti-TNF (open circles) treated cohorts. As expected, synovial areas and talus bone volumes were significantly correlated in the combined treatment groups (R^2^ = 0.50, *p < 0.001*) suggesting that reduction of talus bone volumes and synovitis with anti-TNF therapy are related. However, these outcomes were not correlated within the treatment groups, which may represent a limitation of the distinct regional analysis with histology, or the measure of synovial area not being specific to the talus **(A)**. For the knee, the synovial areas and patella bone volume were not correlated in the combined or separated treatment cohorts. The discordance between these outcomes may similarly relate to the limited regional analysis by histology, or the lack of specificity to the patellofemoral joint **(B)**. Statistics: Linear regression analysis with Pearson’s coefficient of determination (R^2^) and *p*-value provided for the placebo and anti-TNF groups combined (All) or separately; **** p < 0.001* or no significance (ns) *p > 0.05*. Sample size: Each datapoint represents an individual limb from TNF-Tg placebo and TNF-Tg anti-TNF cohorts (n = 5 mice, n = 10 limbs per group).


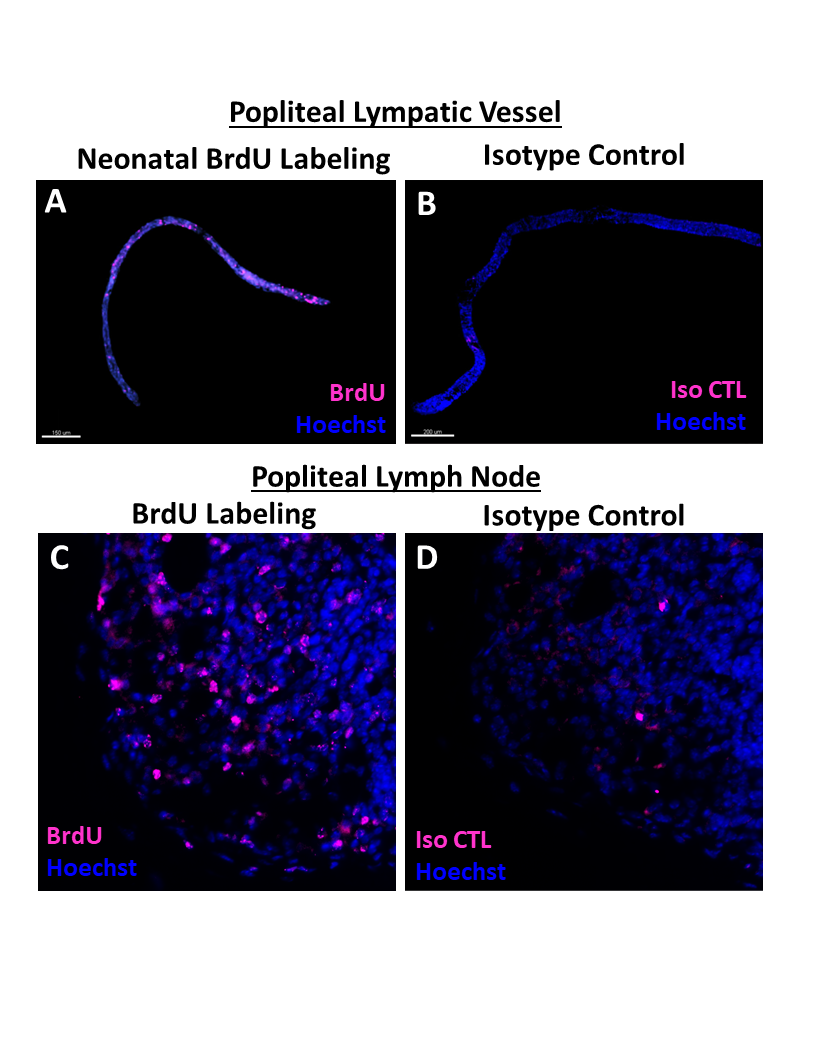


**Supplementary Figure 3. Validation of the BrdU labeling protocol.** To confirm the effective *in vivo* BrdU labeling protocol as we previously described^26^, we administered BrdU daily (i.p., 0.1 mg/g) to neonatal WT mice starting at post-natal day 21 (P21) for 1-2 weeks. PLVs were then harvested, and immunostained for αSMA and BrdU (pink) with a Hoechst nuclear stain (blue). Similar to Figure 4, confocal stacks across the entire length and depth of the whole-mounted PLVs were collected and the proportion of αSMA^+^/BrdU^+^ nuclei was quantified (n = 2 mice, n = 6 PLVs) with a representative image of a PLV from a neonatal mouse following 2-weeks of BrdU administration. The neonatal WT PLVs (BrdU from P21 to P28/35) exhibited a significantly increased LMC turnover rate of 21.4±15.0% per week compared to the PLVs from adult WT and TNF-Tg mice (BrdU from 8- to 9.5-months of age) (0.58±1.1% (WT), 0.71±0.79% (TNF-Tg placebo), and 0.48±0.50% (TNF-Tg anti-TNF) BrdU^+^ LMCs per week, ***** p < 0.0001* neonatal vs all other groups, one-way ANOVA with Tukey’s multiple comparisons) **(A)**. A representative image of an isotype control stained PLV (n = 2 total; 1 WT, 1 anti-TNF) is shown, where 0.17±0.02% stained cells per week may be attributed to non-specific staining **(B)**. We also confirmed the *in vivo* BrdU labeling was effective in the WT and TNF-Tg treatment groups by investigating BrdU staining (pink) in paraffin-embedded sections of the associated PLNs (n = 4 WT, n = 1 TNF-Tg placebo, and n = 3 TNF-Tg anti-TNF PLNs). A representative image of a PLN from an anti-TNF treated TNF-Tg mouse is shown with a remarkable number of BrdU^+^ cells **(C)** relative to the isotype staining control of the adjacent histological section **(D)** demonstrating the effectiveness of the BrdU labeling.


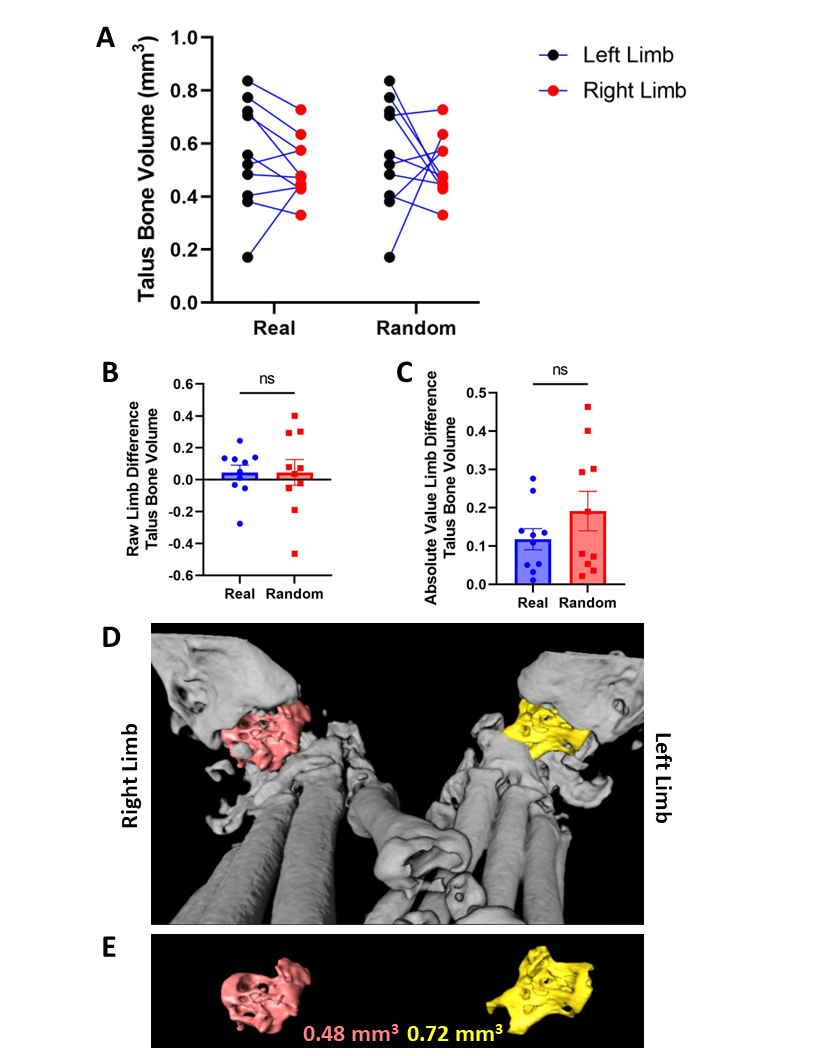


**Supplementary Figure 4. Asymmetric erosive ankle arthritis in TNF-Tg mice.** To quantitatively validate the asymmetry in TNF-Tg arthritis as noted as previous studies^9,10,12,13,15-17^, we evaluated the talus bone volumes in all TNF-Tg mice from this study at 8-months-old prior to treatment initiation (n = 10 mice). As the asymmetry of disease promotes the use of individual limbs as independent variables for analysis, we compared the “Real” pairs with “Randomized” pairs, which represents true limb independence. When comparing talus bone volumes for the real and randomized limb pairings, we found no significant interaction between left vs right limb pairs (*p > 0.05*, 2-Way ANOVA), which suggests that the differences between left and right limbs in real pairings is the same as randomization **(A)**. We also evaluated the raw change in talus bone volume between left and right limbs within the real vs randomized pairs and found no significant difference (*p > 0.05*, unpaired t-test) in the distribution of values without lateralization, supporting that left/right bone sizes are randomly distributed **(B)**. When evaluating the absolute value of the left vs right bone volume difference, we similarly find no significant difference in the distribution of values between real and randomized (*p > 0.05*, unpaired t-test), and both groups differ remarkably from 0, the value indicating true symmetry **(C)**. A representative image of the ankles from a single TNF-Tg mouse with talus bones colored (pink = right, yellow = left) **(D)**, and demonstrates the dramatic asymmetry in erosive disease that occurs in individual animals **(E)**. For these reasons, individual limbs serve as independent variables for quantitative analysis.
